# Three dimensional-stacked complementary thin-film transistors using n-type Al:ZnO and p-type NiO thin-film transistors

**DOI:** 10.1038/s41598-018-22430-6

**Published:** 2018-03-05

**Authors:** Ching-Ting Lee, Chia-Chi Chen, Hsin-Ying Lee

**Affiliations:** 1 Institute of Microelectronics, Department of Electrical Engineering, National Cheng Kung University, Tainan, 701, Taiwan, Republic of China; 2 Department of Photonics Engineering, Yuan Ze University, Taoyuan 320, Taiwan, Republic of China; 3 Department of Photonics, National Cheng Kung University, Tainan 701, Taiwan, Republic of China

## Abstract

The three dimensional inverters were fabricated using novel complementary structure of stacked bottom n-type aluminum-doped zinc oxide (Al:ZnO) thin-film transistor and top p-type nickel oxide (NiO) thin-film transistor. When the inverter operated at the direct voltage (V_DD_) of 10 V and the input voltage from 0 V to 10 V, the obtained high performances included the output swing of 9.9 V, the high noise margin of 2.7 V, and the low noise margin of 2.2 V. Furthermore, the high performances of unskenwed inverter were demonstrated by using the novel complementary structure of the stacked n-type Al:ZnO thin-film transistor and p-type nickel oxide (NiO) thin-film transistor.

## Introduction

Recently, in view of the inherent advantages of the direct wide bandgap energy and the high radiation hardness, the transparent metal oxide materials were widely used in electronic devices^1,2^, optoelectronic devices^3,4^, and sensors^5,6^. To prevent the influence of the generated current induced by the visible light, the transparent metal oxide materials were used for fabricating thin-film transistors (TFTs) and applying in display systems^7–11^. In the past decades, the transparent p-type and n-type thin-film transistors were respectively demonstrated and applied in various systems^12–14^. To simplify integrated circuit design and reduce power consumption, the complementary electronic devices become the basic structure in practical circuits. In the recent years, the complementary thin-film electronic devices and the hybrid complementary metal-oxide-semiconductor thin-film devices were reported, previously^15,16^. Furthermore, the three-dimensionally stacked n-type a-In-Ga-Zn-O and p-type poly-(9,9-dioctylfluorene-co-bithiophene) complementary thin-film transistor was reported^17^. Since the nickel oxide (NiO) thin films exhibit transparent p-type properties and have high bandgap energy from 3.6 eV to 4.0 eV, they have been used in organic and dye-sensitized solar cells^18^, organic light-emitting diodes^19^, and electrochemical devices^20^. Furthermore, because the aluminum-doped zinc oxide (Al:ZnO) thin films exhibit transparent n-type properties and have high bandgap energy from 3.2 eV to 3.6 eV, they have been used in transparent electrodes^21^, electrooptical devices^22^, and electronic devices^23^. In this work, the complementary thin-film transistors (CTFTs) were constructed by p-type TFTs and n-type TFTs fabricated using the NiO channel layer and the Al:ZnO channel layer, respectively. Moreover, to minimize the device area, the three-dimensional stacked structure of the CTFTs was proposed and applied as inverters.

## Results

### Characteristics of n-type Al:ZnO TFTs and p-type NiO TFTs

**Figure 1:**
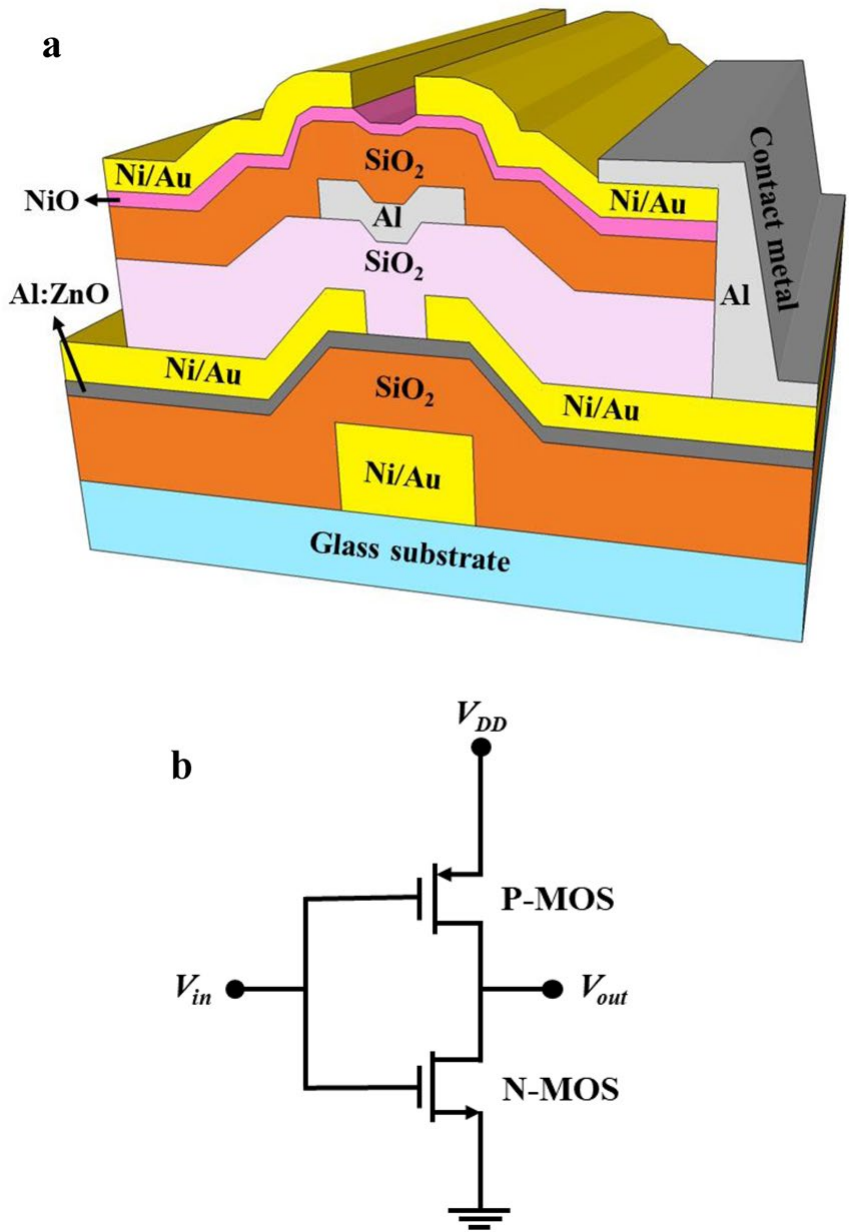
(**a**) Schematic diagram of the three dimensional-stacked complementary thin-film transistors inverter and (**b**) circuit.

**Figure 2:**
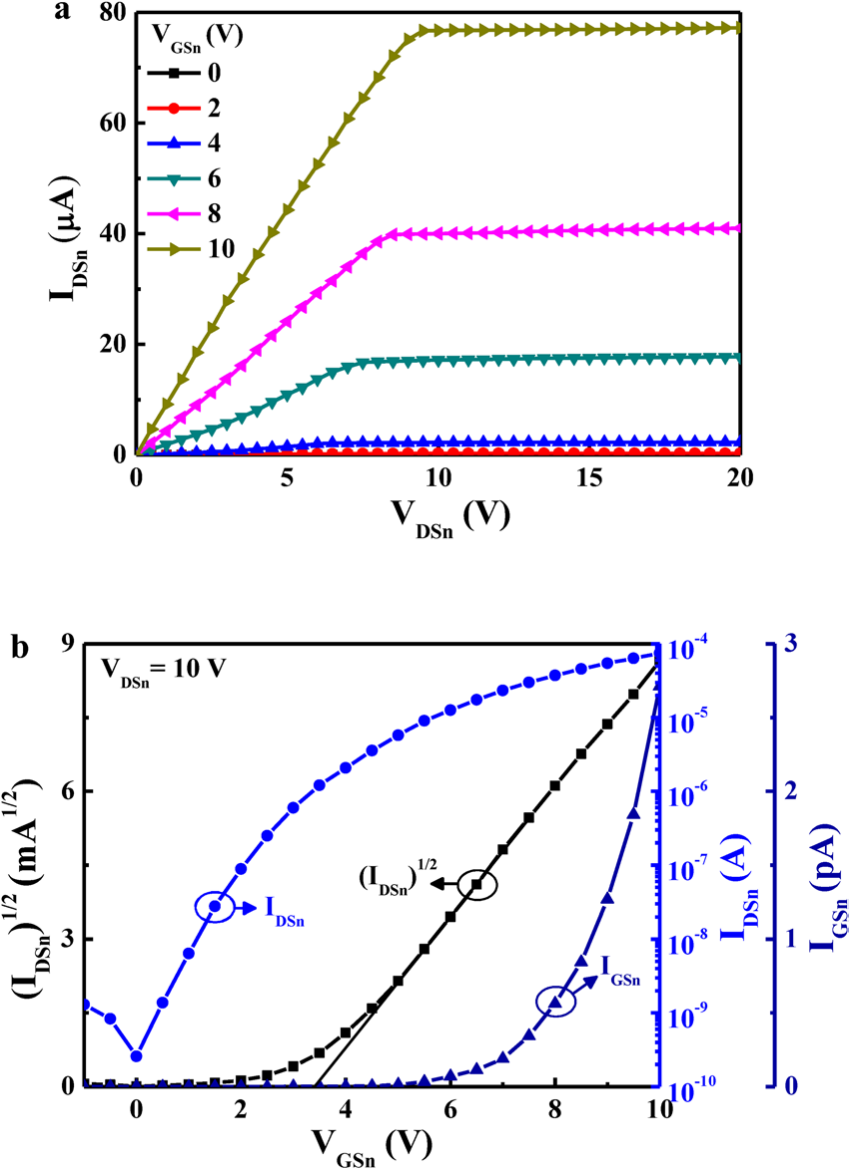
(**a**) Drain-source current-drain-source voltage characteristics and (**b**) drain-source current and transconductance as a function of gate-source voltage of bottom n-type Al:ZnO thin-film transistors.

In the three dimensional-stacked CTFT inverter, the top p-type NiO TFT was stacked on the bottom n-type Al:ZnO TFT as shown in Fig. 1(a). The schematic diagram of the three dimensional-stacked complementary thin-film transistor inverter circuit was shown in Fig. 1(b). To study the characteristics, the bottom n-type Al:ZnO TFTs and the top p-type NiO TFTs were respectively measured using an Agilent 4156 C semiconductor parameter analyzer at a room temperature. Figure 2(a) shows the dependence of the drain-source current (I_DSn_) on the drain-source voltages (V_DSn_) of the bottom n-type Al:ZnO TFTs operated at various gate-source voltages (V_GSn_). It was found that the associated saturation drain-source current (I_DSSn_) at a V_DSn_ of 10 V and a V_GSn_ of 10 V was 77 μA. In general, the drain-source current (I_DS_) as a function of the gate-source voltage (V_GS_) of TFTs operated at the saturation region can be expressed as:

1 $${I}_{DS}=\frac{W{\mu }_{FE}{C}_{ox}}{2L}{({V}_{GS}-{V}_{TH})}^{2}$$

where μ_FE_ is the effective field-effect mobility, C_ox_ is the capacitance per unit gate insulator area, V_TH_ is the threshold voltage, W and L are the channel width and the channel length, respectively. According to the measured I_DSn_-V_DSn_ characteristics, the (I_DSn_)^½^ and the I_GSn_ as a function of the V_GSn_ of the bottom n-type Al:ZnO TFTs operated at V_DSn_ = 10 V were shown in Fig. 2(b). By plotting the (I_DSn_)^½^ versus V_GSn_ and extrapolating the linear line to the V_GSn_ axis, the intercept value is the associated threshold voltage. It could be found that the threshold voltage of the bottom n-type Al:ZnO TFTs was 3.4 V. When the TFTs operated at V_DSn_ of 10 V and V_GSn_ of 10 V, the associated gate leakage current and the on-to-off current ratio were 2.7 pA and 2.9 × 10^6^, respectively. By defining the subthreshold swing (S) as S = dV_GS_/d (log I_DS_), the S value of the bottom n-type Al:ZnO TFTs was 0.78 V/decade.

**Figure 3:**
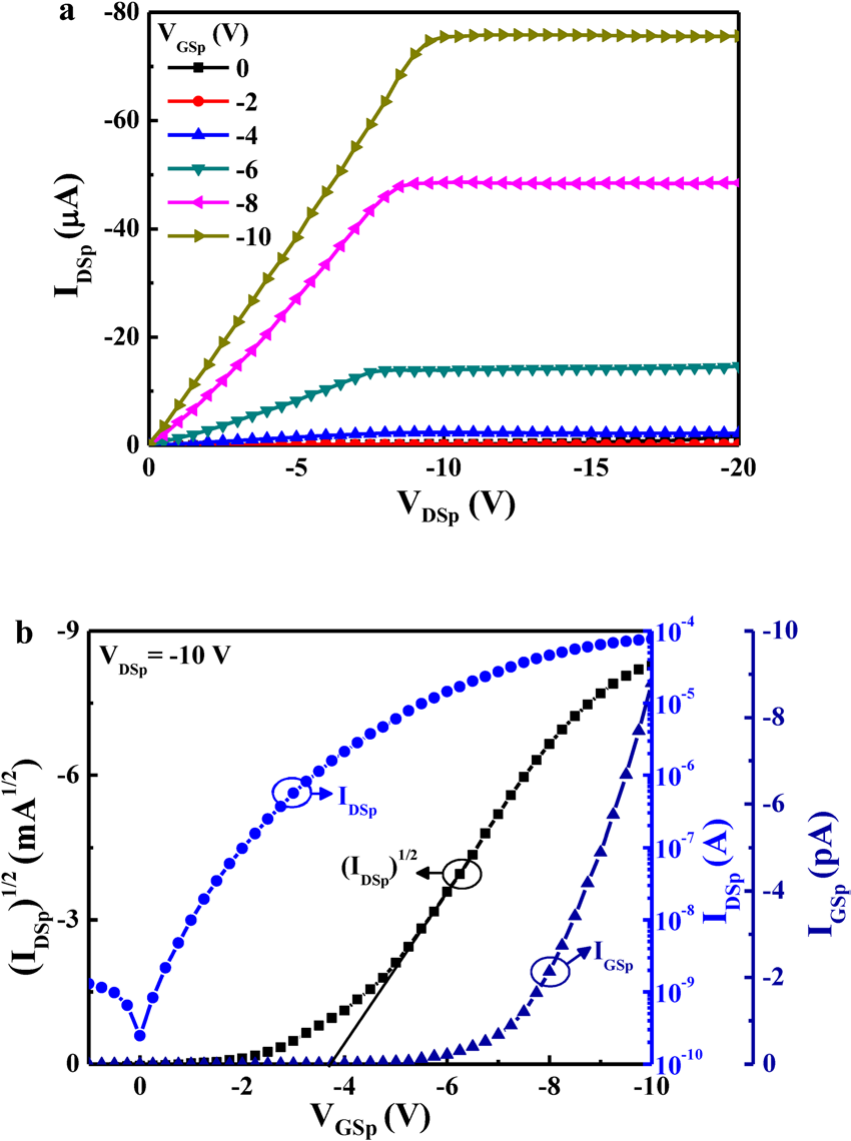
(**a**) Drain-source current-drain-source voltage characteristics and (**b**) drain-source current and transconductance as a function of gate-source voltage of top p-type NiO thin-film transistor.

The measured I_DSp_-V_DSp_ characteristics of the top p-type NiO TFTs operated at various V_GSp_ were shown in Fig. 3(a). Furthermore, the associated (I_DSp_)½ and I_GSp_ as a function of the V_GSp_ were shown in Fig. 3(b). It was found that the saturation drain-source current (I_DSSp_) and the gate leakage current were −77 μA and −8.9 pA, respectively, when the top p-type Ni TFTs operated at a V_DSp_ of −10 V and a V_GSp_ of −10 V. The associated on-to-off current ratio was 1.5 × 10^6^. The associated threshold voltage and subthreshold swing were −3.7 V and 0.56 V/decade, respectively. Table 1 listed the performance summary of the n-type Al:ZnO TFTs and the p-type NiO TFTs.

**Table 1:** Performance summary of n-type Al:ZnO TFTs and p-type NiO TFTs.

Performance	Saturation current	Gate leakage current	Threshold voltage	On-to-off current ratio	Subthreshold swing
Device					
n-type Al:ZnO TFTs	77 μA	2.7 pA	3.4 V	2.9×10^6^	0.78 V/decade
p-type NiO TFTs	–77 μA	–8.9 pA	–3.7 V	1.5×10^6^	0.56 V/decade

### Three dimensional stacked inverters of complementary thin-film transistors

**Figure 4:**
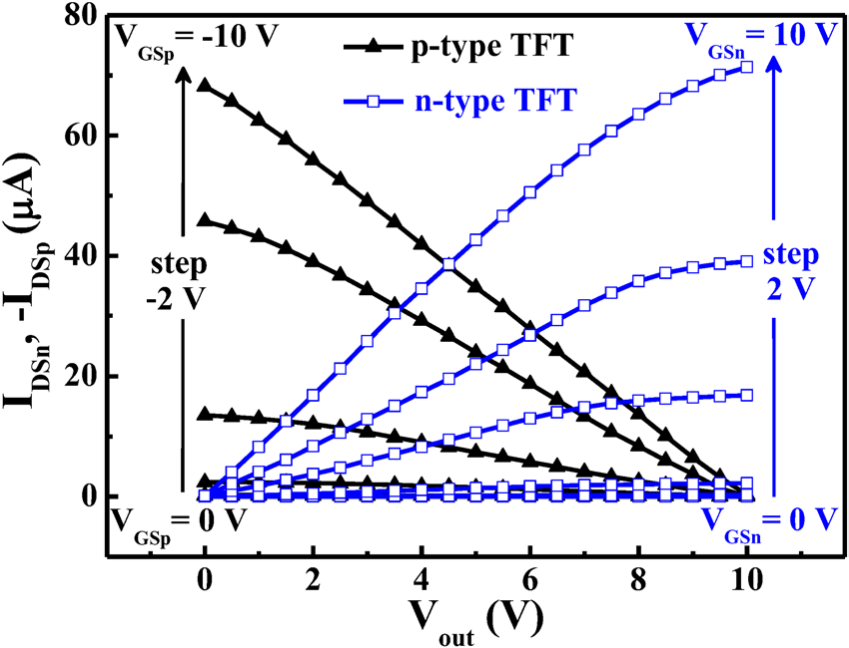
Load line characteristics of three dimensional-stacked complementary thin-film transistors inverters operated at a V_DD_ of 10 V.

**Figure 5:**
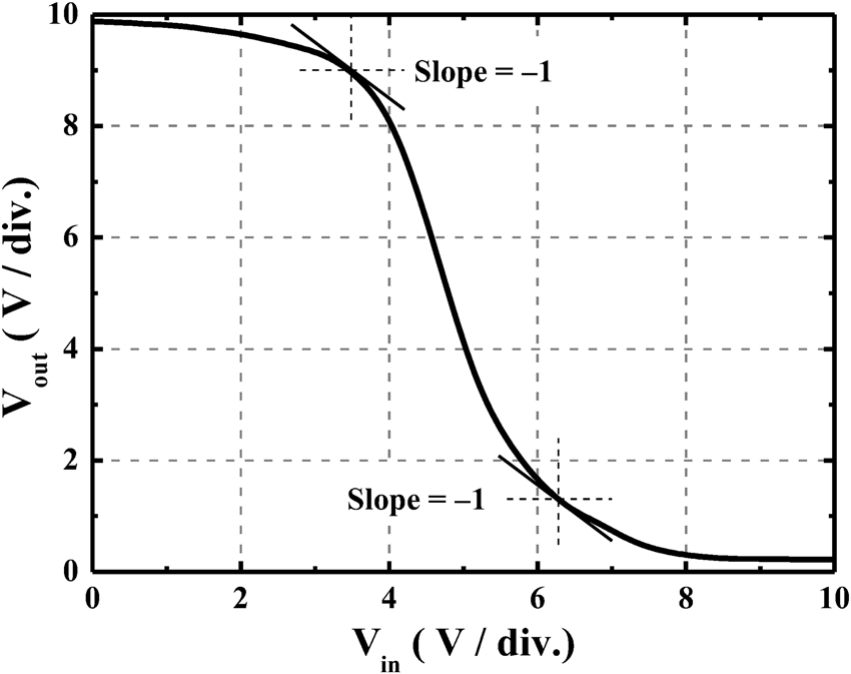
Static transfer characteristics of the three dimensional-stacked complementary thin-film transistors inverter operated at V_DD_ of 10 V and input voltage from 0 V to 10 V.

Using the above-mentioned inverter of the stacked bottom n-type Al:ZnO TFT and top p-type NiO TFT shown in Fig. 1(a) and (b), Fig. 4 shows the load line characteristics of the inverters operated at a V_DD_ of 10 V and an input voltage from 0 V to 10 V. The quiescent point of the inverter located at the intersection point of the load line characteristics of the n-type TFT and the p-type TFT. As shown in Fig. 1(b), when the input voltage of the inverter was 0 V, the bottom n-type Al:ZnO TFT (driver) operated at the cutoff region due to the V_GSn_ = 0 V. Consequently, the highest output voltage (V_OH_) of the inverter was eventually equal to 10 V. When the input voltage increased, the V_GSn_ increased and the |V_GSp_| = |V_GSn_ − V_DD_| decreased. Consequently, the I_DSn_ increased and the |I_DSp_| deceased. As shown in Fig. 4, since the I_DSn_ was equal to the |I_DSp_|, it was worth to note that the output voltage was forced to be decreased. When the input voltage V_in_ = V_GSn_ was 10 V (i.e V_GSp_ = 0 V), the lowest output voltage (V_OL_) was 0.1 V. Since the V_OH_ and V_OL_ of the inverter was 10 V and 0.1 V, the corresponded output swing (V_OH_ − V_OL_) was 9.9 V. Figure 5 shows static V_out_ − V_in_ transfer characteristics of the inverter operated at the V_DD_ of 10 V and the input voltage from 0 V to 10 V. The input high voltage (V_IH_) and the input low voltage (V_IL_) were defined as at the point with the slope of −1 in Fig. 5. When the high noise margin (NM_H_) was defined as V_OH_ − V_IH_, and the low noise margin (NM_L_) was defined as V_IL_ − V_OL_, the NM_H_ and NM_L_ of the inverter were 2.7 V and 2.2 V, respectively. As shown in Fig. 5, when the output voltage V_out_ = V_DD_/2, the operated input voltage V_in_ was 4.9 V, which very closed to the V_out_ = V_DD_/2 = 5 V. This experimental result indicated that the three dimensional stacked complementary thin-film transistors could work as an unskewed inverter.

**Figure 6:**
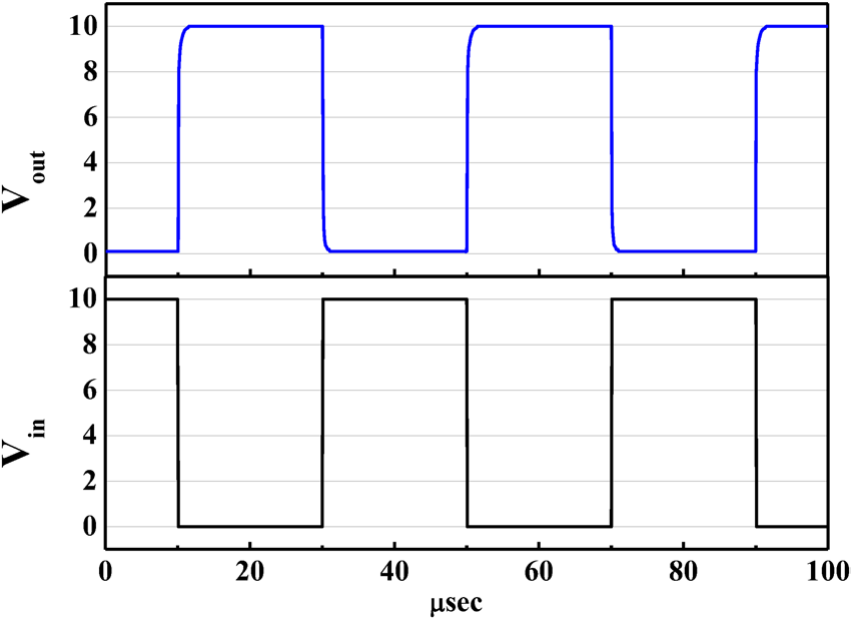
Time domain response of output voltage as a function of input pulse voltage applied to inverter.

When an input pulse voltage from 0 V to 10 V was applied to the inverter operated at V_DD_ = 10, the output voltage was shown in Fig. 6. It was found that the output voltage could quite response with the input pulse voltage. The response time of the output voltage was about 2 μs. It was expected that the inverter could be operated at 500 kHz.

## Discussion

In this work, the inverter was constructed using the three dimensional stacked bottom n-type Al:ZnO TFTs and top p-type NiO TFTs. In the inverter, the n-type TFTs and the p-type TFTs worked as the driver and the load, respectively. When the inverter operated at the V_DD_ of 10 V and the input voltage from 0 V to 10 V, the performances of unskewed inverter were resulted. Furthermore, the output swing voltage of 9.9 V, the high noise margin of 2.7 V and the low noise margin of 2.2 V in the inverter were obtained. Since the three dimensional stacked structure could minimize the area of the complementary thin-film transistors, it would be the promising candidate structure in systems. As our best knowledge, the three dimensional stacked structure is the first reported three dimensional complementary thin-film transistors.

When the SiO_2_ insulator and the Ni, Au and Al metals used in the inverters were replaced using transparent metal oxide insulator and the transparent conducting metal oxide electrode, the transparent n-type and p-type TFTs could be obtained. Consequently, the transmittance of the resulting TFTs could be improved. Since the p-type ZnO TFTs were previously demonstrated^12^, the total ZnO-based CTFTs could be achieved by replacing the p-type NiO TFTs using the p-type ZnO TFTs. Furthermore, in view of the high performance and the high stability TFTs using ZnO-based materials, such as quaternary indium gallium zinc oxide^24^ and quinary indium gallium zinc aluminum oxide^25^, the performances of the CTFTs could be further improved by using those materials as the channel layer of TFTs. Because the flexible devices became prevalent candidate in application of systems, the CTFTs were fabricated on flexible substrates would be a promising study topic. In the display system, the transparent TFTs can replace the conventional shadowy TFTs to improve the transparency and aspect of pixel. Furthermore, if the complementary thin-film transistors were used to replace the convention TFTs, the switch performance and the power consumption of pixel were improved. To reduce the occupied area of TFTs in pixel, the stacked complementary thin-film transistors could reduce the occupied area compared with that of the planar-structured complementary thin-film transistors. The complementary device was the basic structure of integrated circuits. Therefore, the proposed stacked complementary thin-film transistors studied in this work can be expected to be used in three dimensional integrated circuits and reduced the occupied area of the complementary thin-film transistors.

## Method

### Preparation of inverter using stacked n-type Al:ZnO TFTs and p-type NiO TFT

To fabricate the bottom n-type Al:ZnO TFT on glass substrate, an AZ6112 photoresist was spread on glass substrates. The gate window (gate length = 30 μm and gate width = 200 μm) was opened by a standard photolithography technique. The gate metals of Ni/Au (20 nm/70 nm) were deposited using an electron beam evaporator and formed using a lift off process. After depositing a 140-nm-thick SiO_2_ insulator layer using a radio frequency (RF) magnetron cosputter, a 30-nm-thick Al:ZnO channel layer of the bottom n-type TFTs was deposited using the RF magnetron cosputter system with dual targets of Al target and ZnO target. The Al:ZnO channel layer was deposited with the sputtered RF power of 100 W applied to the ZnO target and the sputtered RF power of 30 W applied to the Al target under an argon flow rate of 30 sccm and a working pressure of 75 mtorr. Using the Hall measurement at room temperature, the electron concentration and the electron mobility of the Al:ZnO layer were 8.3×10^16^ cm^−3^ and 12.2 cm^2^/V-s, respectively. When the AZ6112 photoresist was spread on the sample, the source window and the drain window were opened by the standard photolithography technique. The distance between the source window and the drain window was 10 μm. The width of both the windows was 100 μm. The source metals and the drain metals of Ni/Au (20 nm/70 nm) were deposited using the electron beam evaporator and formed using the lift off process. To form ohmic contact, the sample was annealed in a pure nitrogen ambient furnace at 200 °C for 3 minutes. Furthermore, the RF magnetron cosputter was used to deposit a 250-nm-thick SiO_2_ insulator layer to separate the bottom TFT and the followed top TFT in the three-dimensional stacked CTFT inverter.

**Figure 7:**
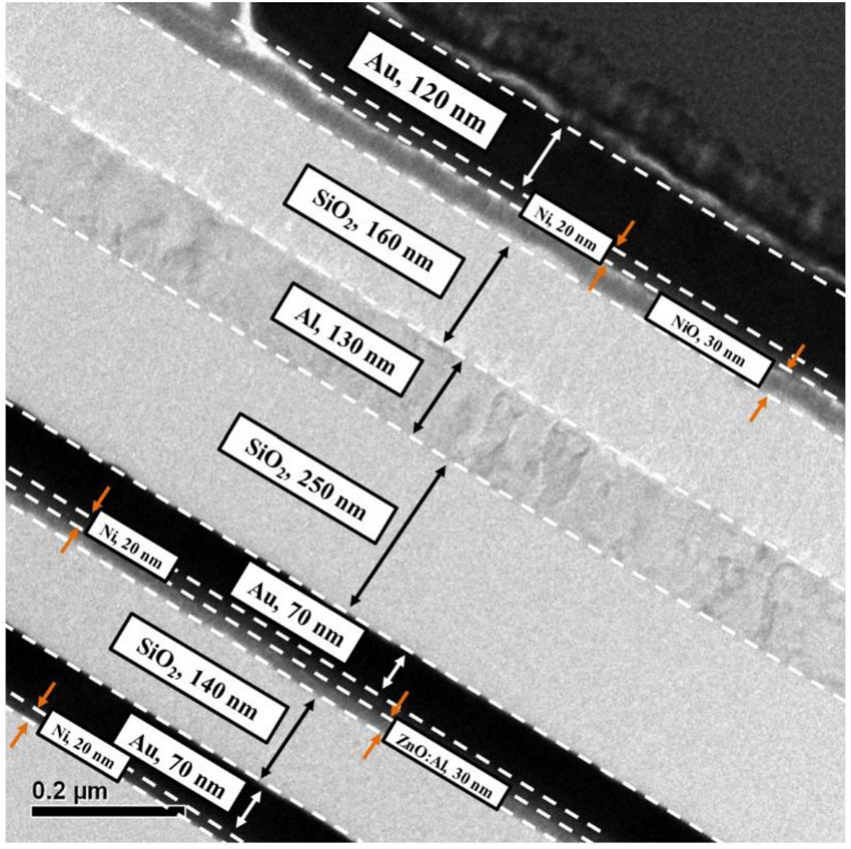
Transmission electron microscope image of the extended cross-sectional view of the inverter.

The fabrication process and the dimension of the stacked top p-type NiO TFTs were the same as the above-mentioned bottom n-type Al:ZnO TFTs. However, in the top p-type NiO TFTs, the 130-nm-thick Al metal and the 30-nm-thick NiO layer were used as the gate electrode and the channel layer, respectively. The 160-nm-thick SiO_2_ insulator layer was deposited between the Al gate electrode and the NiO channel layer using RF magnetron cosputter. The NiO layer was deposited by the RF magnetron sputter with sputtered RF power of 125 W applied to the Ni target under working pressure of 10 mtorr and N_2_/O_2_ flow rate of 10 sccm/40 sccm. Using the Hall measurement at room temperature, the hole concentration and the hole mobility of the p-type NiO layer were 1.81×10^16^ cm^−3^ and 2.22 cm^2^/V-s, respectively. To form the source and drain electrodes of the p-type NiO TFTs, the Ni/Au (20 nm/ 120 nm) metals were deposited using an electron beam evaporator and patterned using a lift-off process. After fabricating the top p-type NiO TFT stacked on the bottom n-type Al:ZnO TFT worked as an inverter, the input port was formed by interconnecting the gate electrode of the p-type NiO TFT with the gate electrode of the n-type Al:ZnO TFT. Moreover, the output port of the inverter was formed by interconnecting the drain electrode of the p-type NiO TFT with the drain electrode of the p-type Al:ZnO TFT. After spreading AZ6112 photoresist on the front side of the top p-type NiO TFTs, the windows of the surrounding region of the drain electrode and the gate electrode were opened using the standard photolithography technique. After the materials under windows were etched, a thick Al metal was deposited as the interconnection metal by an electron beam evaporator. Figure 7 shows the transmission electron microscope image of the extended cross-sectional view of the inverter.
